# Gene and microRNA modulation upon trabectedin treatment in a human intrahepatic cholangiocarcinoma paired patient derived xenograft and cell line

**DOI:** 10.18632/oncotarget.13575

**Published:** 2016-11-24

**Authors:** Caterina Peraldo Neia, Giuliana Cavalloni, Giovanna Chiorino, Paola Ostano, Massimo Aglietta, Francesco Leone

**Affiliations:** ^1^ University of Turin Medical School, Department of Oncology, IRCCS-Institute Candiolo, Italy; ^2^ Medical Oncology Division, Fondazione del Piemonte per l’Oncologia (FPO), IRCCS-Institute Candiolo, Italy; ^3^ Cancer Genomics Laboratory, Fondazione Edo ed Elvo Tempia Valenta, Biella, Italy

**Keywords:** trabectedin, Intrahepatic cholangiocarcinoma, microarray, microRNA, silencing

## Abstract

Intrahepatic cholangiocarcinoma (ICC) is an aggressive and lethal malignancy with limited therapeutic options. Trabectedin has a high antitumor activity in preclinical models of biliary tract carcinoma (BTC), being a promising alternative treatment. Here, we studied the effect of trabectedin at transcriptomic level on an ICC patient derived xenograft (PDX) and on the derived cell line, MT-CHC01. Further, putative targets of trabectedin were explored in the *in vitro* model. *In vitro*, trabectedin inhibited genes involved in protein modification, neurogenesis, migration, and motility; it induced the expression of genes involved in keratinization, tissues development, and apoptotic processes. In the PDX model, trabectedin affected ECM-receptor interaction, focal adhesion, complement and coagulation cascades, Hedgehog, MAPK, EGFR signaling via PIP3 pathway, and apoptosis. Among down-regulated genes, we selected SYK and LGALS1; their silencing caused a significantly reduction of migration, but did not affect proliferation in *in vitro* models. In MT-CHC01 cells, 24 microRNAs were deregulated upon drug treatment, while only 5 microRNAs were perturbed by trabectedin in PDX. The target prediction analysis showed that SYK and LGALS1 are putative targets of up-regulated microRNAs. In conclusion, we described that trabectedin affected genes and microRNAs involved in tumor progression and metastatic processes, reflecting data previously obtained at macroscopically level; in particular, we identified SYK and LGALS1 as new putative targets of trabectedin.

## INTRODUCTION

Intrahepatic cholangiocarcinoma (ICC) is the second most common liver cancer type [1, 2]. It is aggressive and with poor prognosis, with limited therapeutic options. Patients with unresectable disease (70-90%) have a poor prognosis with a survival of less than 12 months following diagnosis [3, 4]. Conventional therapy gives only marginal benefit [5]. Several dosage schedules of single-agent gemcitabine (gem) have been tested obtaining response rates of about 20%. To date, the standard of treatment is the combination of gem and cisplatin; a meta-analysis conducted by Valle and collaborators demonstrated that this association gave a significant survival advantage, compared to gem alone (progression free survival, PFS, of 8 vs. 6.7 months; overall survival, OS of 11.6 months vs 8.0 months) [6]. These data demonstrate the lack of effective therapies and consequently the need of identifying alternative therapeutic approaches.

Trabectedin (Ecteinascidin-743 or ET-743) is a marine derived compound isolated from the Caribbean tunicate Ecteinascidia Turbinata with a potent antitumor effect, based on a particular mechanism of action. It acts as an alkylating agent by binding to the minor groove of DNA, inducing double-strand DNA breaks and interacting with transcription factors, in particular the NF-Y which interacts with the CCAAT box [7–10]. Recent data suggest that the nucleotide excision repair mechanism plays a key role in trabectedin antitumor activity. It was described that trabectedin could specifically interact with the DNA transcription-coupled nucleotide excision repair (TC-NER) process [11], which in normal conditions repairs DNA adducts caused by UV rays, cisplatin or others anticancer agents by involvement of proteins belonging to family of XP11 and NER factors. Thus, the double-strand DNA breaks formed are more persistent if there is loss of homologous DNA repair [12]. Further, it has been described that trabectedin had a potent antitumor activity both on tumor cells as well as on microenvironment [13]. It is also known that trabectedin causes a marked decrease in the production of several cytokines and chemokines secreted by monocytes/macrophages and tumor cells. For example, trabectedin treatment down-regulates the expression of IL-6 (Interleukin-6), CCL2, CXCL8, Angiopoietin 2 or VEGF (Vascular endothelial growth factor) [14].

Trabectedin has been approved for treatment of ovarian cancer and soft-tissue sarcoma with significant activity in liposarcomas, leiomyosarcomas, and Ewing sarcoma, both as a single agent and in combination with other drugs [15–18]. Trabectedin has also shown promising pre clinical activity against other different cancer cell lines *in vitro* and transplantable *in vivo* human tumor xenografts [19–28].

We have recently demonstrated that trabectedin has an antitumor activity both *in vitro* and *in vivo* in preclinical models of human biliary tract carcinoma (BTC). We reported that trabectedin inhibits BTC *in vitro* cell growth and causes a significant delay of tumor growth *in vivo* in mouse models of human BTC, EGI-1 extrahepatic cholangiocarcinoma xenograft and a new intrahepatic cholangiocarcinoma (ICC) patient-derived tumor xenograft (PDX) [19], causing a decrease of tumor proliferating cells and tumor vessel formation. Transcriptomic analysis revealed that, upon trabectedin treatment, there is a deregulation of genes involved in IL-6, Sonic Hedgehog and Wnt signaling pathways, all related to cholangiocarcinogenesis.

Here, we used a human intrahepatic cholangiocarcinoma paired PDX and cell line model to investigate the changes in gene and microRNA expression in response to trabectedin. The aim of this study is the identification of differentially expressed genes, of related biological pathways and microRNAs which may be relevant as specific targets of trabectedin. Furthermore, we studied the effective role of two genes as putative targets of trabectedin in the *in vitro* model.

## RESULTS

### Transcriptomic profiles of MT-CHC01 cell line and PDX upon trabectedin treatment

Changes in the expression of various genes were observed upon *in vitro* trabectedin treatment in MT-CHC01 cells. We applied a filter on LogFC value and considered for the analysis only probes with a Log FC <-1 or > 1. Upon trabectedin treatment, 1,254 differentially expressed gene transcripts were identified, of which 948 were down- and 306 up-regulated. Gene Ontology was performed using DAVID Annotation tool; Supplementary Table S1 summarized the biological processes significantly enriched within down-and up-regulated probes. Trabectedin is able to negatively influence regulation of GTPase activity, processes related to neurogenesis, migration, cell adhesion, and microtubules organization. On the other hand, trabectedin induces the expression of genes involved in keratinization, epidermis, endoderm and tissue development, and apoptotic processes.

The same analysis was performed to identify pathways enriched within down- and up-regulated genes (Kegg database) (Supplementary Table S2); the most significant down-regulated pathway includes genes involved in focal adhesion.

The same filter applied to MT-CHC01 was used for PDX data yielding only 28 differentially expressed genes. Relaxing cut-offs (Log FC <0.58 or >0.58 and p-value <0.05), we obtained 1,346 differentially expressed genes of which 628 were down- and 718 up-regulated.

As shown in Supplementary Table S3, Gene Ontology revealed a globally down-regulation of genes involved in eye, organ and skin morphogenesis, tissue development, immune response and regulation of angiogenesis. In contrast, there is an up-regulation of genes involved in inflammation process, muscle development, negative regulation of signal transduction, and different biosynthetic processes.

Further, we found that down-regulated genes caused an enrichment of pathways involved in ECM-receptor interaction, focal adhesion, complement and coagulation cascades, and Hedgehog signaling pathways. On the contrary, for up-regulated genes we found an enrichment of MAPK pathway, EGFR signaling via PIP3, and apoptosis (Supplementary Table S4).

**Figure 1:**
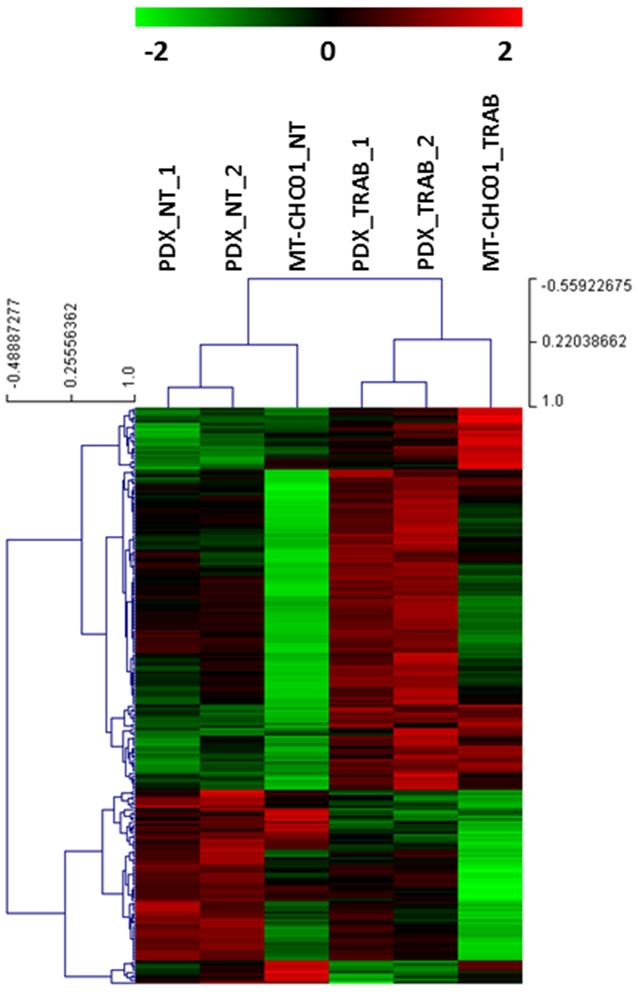
Unsupervised cluster analysis of common modulated genes between *in vitro* and *in vivo* model A clear separation between trabectedin treated and not treated samples is found.

The next step was the identification of common deregulated genes between the *in vitro* and *in vivo* models. We found 223 concordant overlapping genes, of which 75 down-and 148 up-regulated after trabectedin treatment (Supplementary Table S5A-S5B). An unsupervised cluster analysis of this gene set shows a separation between trabectedin-treated from not treated samples (Figure 1).

The Gene Ontology analysis was performed on down-and up-regulated genes separately (Table 1).

**Table 1:** The Gene Ontology analysis on common down- and up-regulated genes

Category (BP-5)	Name	p-value	Expression upon trabectedin
0000988	tissue development	0.000884	down
0009887	organ morphogenesis	0.001	down
0051969	regulation of transmission of nerve impulse	0.002	down
0031644	regulation of neurological system process	0.003	down
0043588	skin development	0.005	down
0010556	regulation of macromolecule biosynthetic process	0.00001	up
0045449	regulation of transcription	0.00003	up
0031326	regulation of cellular biosynthetic process	0.00004	up
0010468	regulation of gene expression	0.00005	
0019219	regulation of nucleobase, nucleoside, nucleotide and nucleic acid metabolic process	0.0001	up
0045596	negative regulation of cell differentiation	0.003	up

BP-5: biological process level 5

The ECM-receptor transition pathway, Systemic lupus erythematosus, Hedgehog signaling pathway, and Basal cell carcinoma (p <0.05) are the common pathways enriched by down-regulated genes. No statistically significant common pathways are enriched by up-regulated genes.

**Figure 2:**
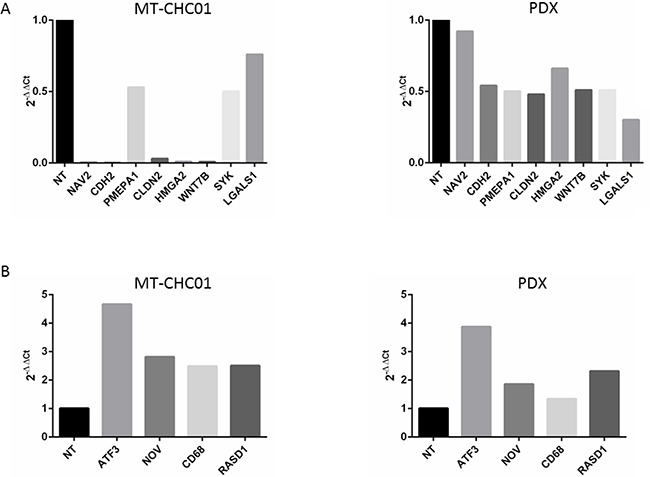
Gene expression validation by qRT-PCR of selected genes altered in microarray analysis **Panel A** shows down-regulated genes and **Panel B** up-regulated genes after trabectedin treatment. X axis: gene expression of MT-CHC01 and PDX, respectively. Y axis: 2-ΔΔCt for qRT-PCR.

In order to validate expression data, we analyzed by RT-qPCR the expression of the common down-regulated CLDN2, CDH2, WNT7B, PMEPA1, NAV2, SYK, LGALS1, and the up-regulated ATF3, NOV, CD68, RASD genes. The same trend of expression was revealed for all genes in both MT-CHC01 and PDX after trabectedin treatment, as shown in Figure 2A-2B.

### SYK and LGALS1 are putative targets of trabectedin

**Figure 3:**
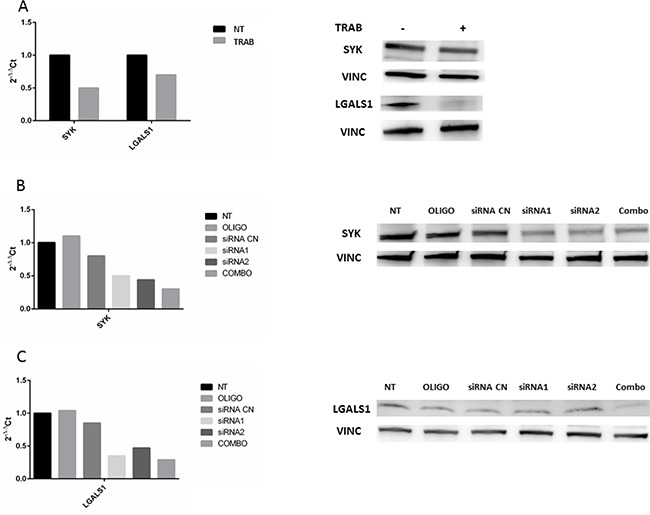
**Panel A**: qRT-PCR quantification and Western Blot analysis of SYK and LGALS1 after trabectedin (5 nM) treatment on MT-CHC01 cells. **Panel B and C**: qRT-PCR quantification and Western Blot analysis of SYK and LGALS1, respectively, after silencing. NT: not treated; OLIGO: cells treated with oligofectamine; siRNA CN: cells treated with negative control siRNA; siRNA1 and siRNA2: two different siRNA for SYK or LGALS; COMBO: combination of siRNA1 and siRNA2.

In order to investigate possible new targets of trabectedin, we selected two genes, SYK and LGALS1, resulted down-regulated by drug treatment in both models. To verify if their silencing exerted the same effect of trabectedin, we silenced them at first in MT-CHC01 cells. Both qRT-PCR and Western Blot analysis, either upon trabectedin treatment or after silencing with single specific siRNA or a pool of them, showed the inhibition of targeted genes (Figure 3).

To investigate the functional role of these genes, we performed cell viability and migration assays. Cell viability of MT-CHC01 cells is significant decreased after trabectedin treatment, with an IC50 value of 1.85 nM (Supplementary Figure S1 Panel A-B). Further, trabectedin is able to block cell cycle in G0/G1 and to induce apoptosis after 48 hours of trabectedin treatment (Supplementary Figure S1, Panel C-D).

**Figure 4:**
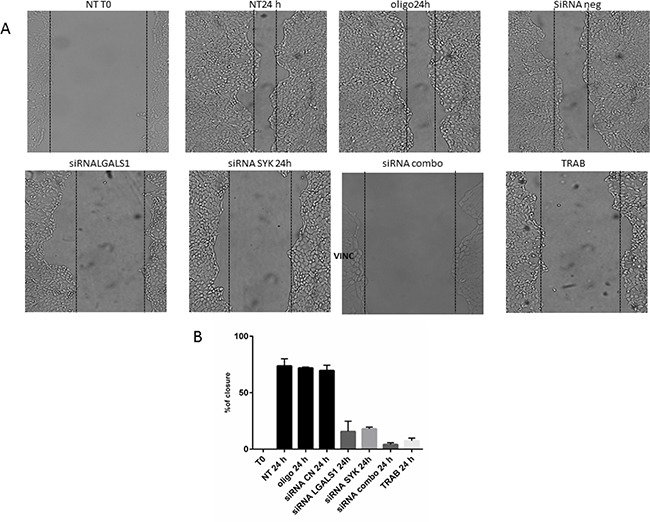
Wound healing assay **Panel A**: representative images of MT-CHC01 wound closure upon different conditions. NT: Not treated; OLIGO: treated with oligofectamine; siRNA CN: treated with negative control siRNA; siRNA LGALS1 and SYK: treated with specific siRNA for LGALS1 and SYK, respectively; siRNA combo: simultaneous treatment with siRNA for LGALS1 and SYK; TRAB: treatment with 5 nM of trabectedin. T0: time of wound. **Panel B**: Statistical analysis of percentage of wound closure; Bars representing the area of closure at different conditions; 100% corresponds to the area at the time of wounding

Conversely, the knock-down of single genes or their combination does not decrease the proliferation of MT-CHC01 cells (data not shown). Cell migration, significantly reduced by trabectedin treatment, is statistically significant inhibited also by silencing of SYK and LGALS1; the simultaneous silencing of target genes potentiates the anti-migratory effect (Figure 4).

**Figure 5:**
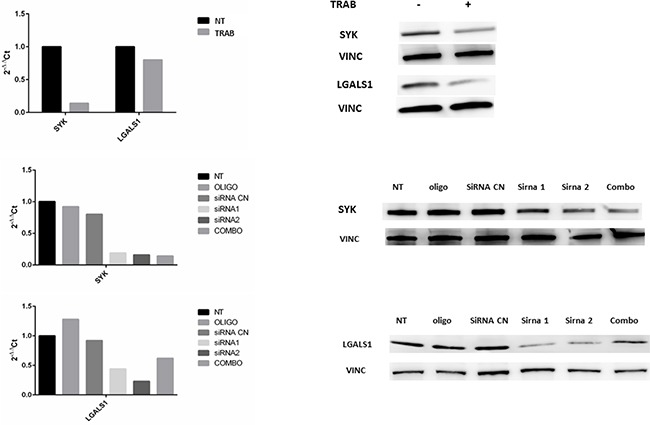
Panel A: qRT-PCR quantification and Western Blot analysis of SYK and LGALS1 after trabectedin (5 nM) treatment on WITT cells **Panel B and C**: qRT-PCR quantification and Western Blot analysis after silencing of SYK and LGALS1. NT: not treated; OLIGO: cells treated with oligofectamine; siRNA CN: cells treated with negative control siRNA; siRNA1 and siRNA2: two different siRNA for SYK of LGALS 1; COMBO: combination of siRNA1 and siRNA2.

To enforce these data, we repeated the same experiments on the ECC cell line WITT. Both qRT-PCR and Western Blot analysis demonstrated that trabectedin inhibited the expression of targets, as well as their silencing (Figure 5).

**Figure 6:**
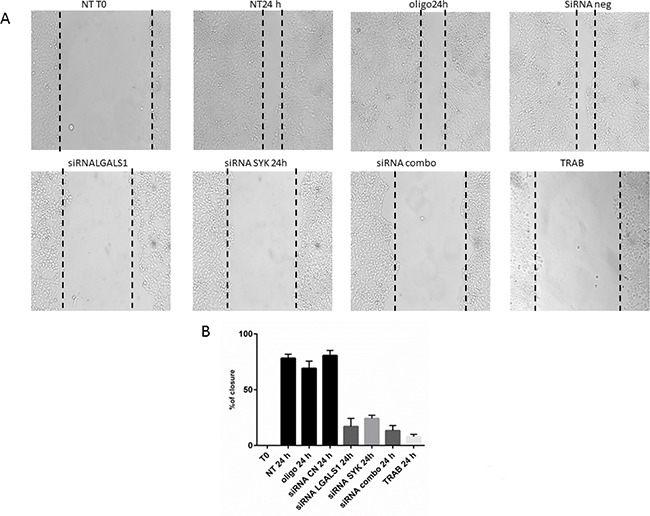
Wound healing assay **Panel A**: representative images of WITT wound closure upon different conditions. NT: Not treated; OLIGO: treated with oligofectamine; siRNA CN: treated with negative control siRNA; siRNA LGALS1 and SYK: treated with specific siRNA for LGALS1 and SYK, respectively; siRNA combo: simultaneous treatment with siRNA for LGALS1 and SYK; TRAB: treatment with 5 nM of trabectedin. T0: time of wound. **Panel B**: Statistical analysis of percentage of wound closure; Bars representing the area of closure at different conditions; 100% corresponds to the area at the time of wounding.

Similarly to the MT-CHC01, single and combined knock-down of SYK and LGALS1 reduced cell migration in a statistical significant manner (Figure 6), but did not have effect on proliferation (data not shown).

### Trabectedin affects the expression of microRNAs involved in apoptosis and tumor progression

**Figure 7:**
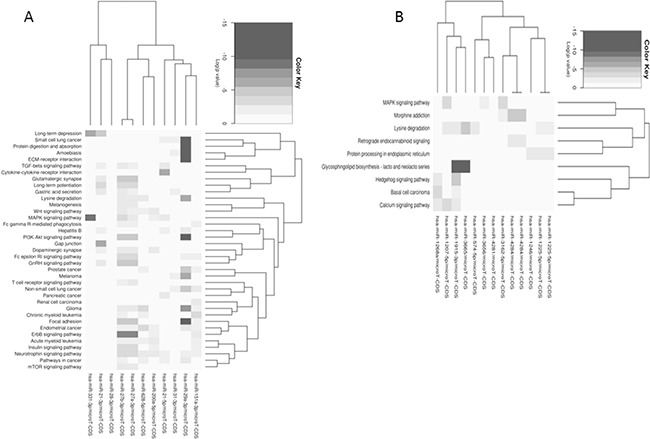
Pathways union analysis of up- **A**. and down- **B**. regulated microRNAs separately upon trabectedin treatment of MT-CHC01 cells.

MicroRNA profiling of MT-CHC01 cells identified 24 deregulated microRNAs upon trabectedin treatment, either up or down-regulated. Supplementary Table S6 summarizes these microRNAs and their known role in cancerogenesis. Globally, upon trabectedin treatment, we found a deregulation of microRNAs involved in tumor progression, proliferation and migration, both as direct or indirect way. In particular, three microRNAs (miR-21-3p, miR-21-5p and miR-31-3p) have already been described as involved and up-regulated in BTC [29–31]; in our setting, they were inhibited by trabectedin, suggesting that they could represent candidate targets of this drug. Using the MiRpath program and the Pathway Intersection algorithm, which provides the pathways affected by the intersection of predicted targets by up/down-regulated microRNAs, we did not find any common pathways for both up-and down-regulated microRNAs. Considering up- and down-regulated microRNAs separately, we performed a pathway union analysis, which used an algorithm able to predict all the significant pathways targeted by selected microRNAs. As shown in Figure 7, down-regulated microRNAs (Panel A) affected in a statistically significant way the focal adhesion, ECM-receptor interaction, protein digestion and absorption, MAPK, PI3K-Akt, ERBB signaling pathways. Considering up-regulated microRNAs (Panel B), we found many affected pathways such as glycosphingolipid biosynthesis, lysine degradation, calcium signaling, MAPK and Hedgehog pathways.

**Figure 8:**
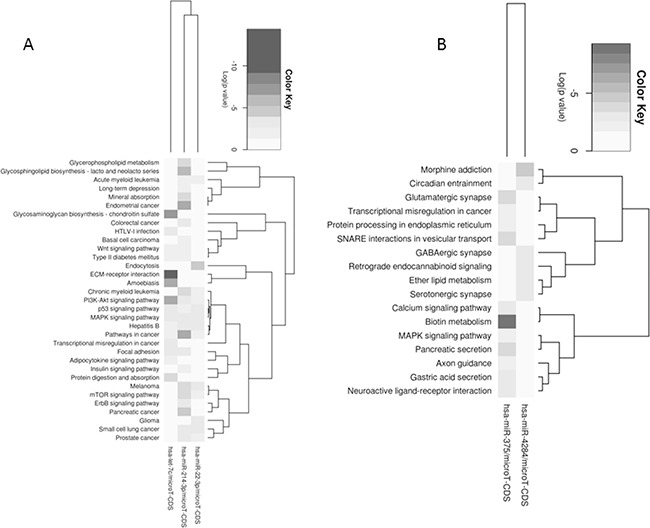
Enriched pathways within up-regulated **A**. and down-regulated **B**. microRNAs upon trabectedin treatment of PDX.

The analysis performed on PDX model showed a deregulation of only five microRNAs, two up-regulated (miR-4284 and miR-375) and three down- (miR-22-3p, let-7c, and miR-214-3p). Figure 8 summarizes pathways affected by down-(Panel A) and up-regulated (Panel B) microRNAs using the Pathway Union algorithm, which evaluated the probability that the examined pathways are significantly enriched for gene targets of at least one microRNA.

Further, we revealed that miR-4284 was up-regulated in both *in vitro* and *in vivo* models after trabectedin treatment. Validation of the microRNAs expression was obtained by RT-qPCR (Supplementary Figure S2).

### MicroRNA-gene targets interaction analysis

**Figure 9:**
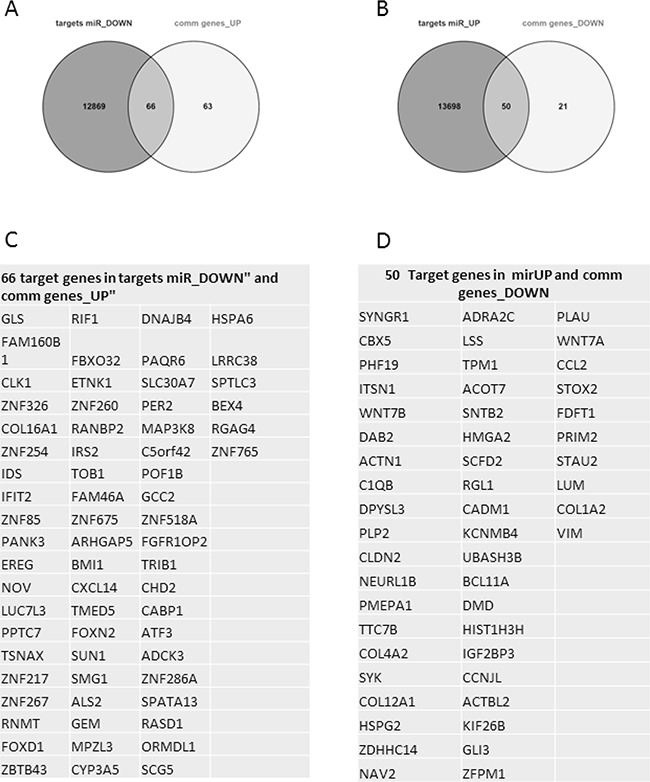
microRNA-target prediction analysis In **Panel A**, Venn diagram showed that 66 up-regulated genes **Panel C** of our signature are predicted targets of down-regulated microRNAs. In **Panel B**, Venn diagram showed that 50 down-regulated genes **Panel D** of our signature are targets of up-regulated micro-RNAs.

To verify if there were interactions among the microRNA signature and the common modulated genes, we used miRWalk 2.0 database. We considered as significant the interactions predicted by at least 4 databases. As shown in Figure 9, we found that up-regulated microRNAs has 13,748 predictive targets, of which 50 down-regulated overlapped to our gene signature. On the contrary, down-regulated microRNAs are able to interact with 12,935 genes, of which 66 up-regulated genes belonged to our dataset.

Interestingly, the microRNA-target prediction analysis showed that SYK is a putative target of all up-regulated microRNAs, while LGALS1 is a predicted target only of miR1207-5p and miR1225-5p.

## DISCUSSION

BTC is an aggressive neoplasia with a complex molecular pathogenesis; the current clinical approaches are limited and the poor prognosis, due also to the scarce therapeutic options, encourages the evaluation of new drugs. Trabectedin is a promising chemotherapeutic agent already used in clinical practice in different cancer types. Our group previously demonstrated that trabectedin has a high antitumor activity in BTC preclinical models, significantly slowing tumor growth, inhibiting proliferation and neovascularization processes, suggesting its potential as alternative chemotherapy option. Here, we studied the global effect of trabectedin on transcriptome in the paired xenopatient-cell line model.

Trabectedin is able to negatively influence the expression of genes involved in migration, invasion, motility, and epithelial to mesenchymal transition (EMT), confirming the important role of trabectedin in inhibiting angiogenesis and metastatic processes demonstrated in other malignancies [19, 28, 32–34]. Interestingly, we observed a down-regulation of genes involved in neurogenesis and regulation of neurological processes; it has been demonstrated that neurons and glial cells not only lead to higher cancer cell proliferation at the tumor invasion front, but also further enhance angiogenesis and neurogenesis in tumors [35].

On the contrary, trabectedin treatment is able to induce the expression of genes involved in p53 pathway, previously described by Martinez and collaborators [36], demonstrating that the drug induced the overexpression of genes related to stress and DNA damage, such as p53, CDKN1A, and ATF3. Moreover, it is able to induce the expression of genes involved in apoptotic and cell death processes, underlying the high therapeutic role of this drug. An overexpression of RASD1 seems to increase apoptosis via mitochondrial apoptosis pathway in prostate, breast, and lung cancer cell lines [37, 38]. IFIT2 is a tumor suppressor gene in gastric cancer [39], promoting apoptosis and cell death. In our models, these genes are induced by trabectedin treatment, supporting the apoptotic process as previously demonstrated *in vitro* [19].

We evidenced that trabectedin perturbes genes involved in different cellular processes, among which the regulation of proliferation and migration. For this reason, we selected two down-regulated genes, SYK and LGALS1 to investigate if their suppression could reflect the effect of trabectedin on these processes. SYK is a non-receptor tyrosine kinase expressed in a variety of tissues, in particular in hematopoietic cells. Its role in cancer progression is controversial; in breast cancer it has a tumor suppressor role, while in head and neck and prostate cancer acted as an oncogene [40–42]. The role of LGALS1, a member of the lectin superfamily, is more defined; it is involved in different cellular function, as transduction, protein-protein interactions, cell-cycle progression, apoptosis, sustained proliferative signaling, resistance to cell death signals, evasion of immune surveillance, induction of angiogenesis, and activation of the metastatic potential. It is over-expressed in colon, liver, pancreatic cancer, in ICC and in their surrounding stroma cells; its overexpression seems to be associated with neoplastic progression and proliferative activities [43–46]. The silencing of each gene separately produced a statistically significant inhibition of migration, but not effect on proliferation. The simultaneous inhibition of these targets potentiates the anti-migratory effect, suggesting a crosstalk between SYK and LGALS1. In the work of Fulcher and collaborators, it has been demonstrated that Gal1, the protein encoded by LGALS1, induced cell activation and migration through the phosphorylation of Syk in dendritic cells [47]. The lack of inhibition of proliferation demonstrated that the silencing of these genes is not sufficient to mimic trabectedin effect, suggesting that a more complex mechanism of drug-targets regulates cell growth. The role of trabectedin on SYK and LGALS1 inhibition is confirmed in the extrahepatic cell line WITT, with a significant reduction of migration ability, supporting our hypothesis that they are putative targets of this drug.

MicroRNA expression revealed a major number of alterations in the *in vitro* model. MiR-21-3p, miR-21-5p and miR-31-3p have already been described in BTC, in particular they are up-regulated in ICC [31]. In our models, after trabectedin treatment they are significantly down-regulated, making them putative targets of the drug. The same mechanism could be supposed for miRNA 331-3p, down-regulated by trabectedin and demonstrated to promote proliferation and EMT in hepatocellular carcinoma [48]. On the contrary, miR-494-3p is up-regulated after drug treatment; in a previous work of Shen and collaborators, they demonstrated that a constitutive over-expression of this miRNA reduced tumor growth, migration and invasion [49]. In the *in vivo* model, only five miRNAs are affected by trabectedin. It has been previously demonstrated that miR-375, found up-regulated in our PDX model, is a tumor suppressor in colon cancer and pancreatic cancer, by inhibiting PI3K/Akt via [50, 51]. A down-regulation of miR-214 upon trabectedin treatment was also found; Li and collaborators demonstrated that its inhibition correlated with a high risk of metastasis increasing the transcription of TWIST. In our model, TWIST is slightly down-regulated, suggesting that miR-214 could have different mechanism of action in BTC [52]. The unique common microRNA up-regulated in both models is miR-4284. Its role is not clear, but in a work of Yang, miR-4284 seems to be overexpressed by bermamine, a natural chemotherapeutic agent, in glioblastoma cells; they demonstrated its role as tumor suppressor, promoting apoptosis in cancer stem cells of glioblastoma [53]. Interestingly, we found that 116 genes of our signature are predicted targets of deregulated microRNAs; in particular, the down-regulated gene SYK is target of all up-regulated microRNAs, while LGALS1 is a putative target of miR-1207-5p, miR-1225-5p. These microRNAs have a role as tumor suppressors; in fact, their up-regulation caused a reduction of motility and invasion in breast and gastric cancers [54, 55], supporting our data on migration in BTC cell lines. These findings suggest further investigations to study their interaction with SYK and LGALS1, both involved in migration process.

In conclusion, we described that trabectedin affects genes and microRNAs involved in processes related to tumor progression and metastasis, reflecting results obtained at macroscopically level in BTC. We identified two trabectedin putative targets involved in motility and migration, supporting the role of trabectedin as anti-metastatic agent. Further studies of gene-microRNA networking and consequent functional studies will be planned to investigate the complex post-transcriptional alterations caused by trabectedin treatment.

## MATERIALS AND METHODS

### Samples and drug treatment

The patient derived xenograft (PDX) was obtained and treated with trabectedin as previously described [19]. Briefly, PDX were intra venous treated with 0.15 mg/Kg/weekly or with drug vehicle for 21 days. The establishment and characterization process of its paired cell line, MT-CHC01, is described in our recent work [56]. The extrahepatic cell line WITT (provided by Dr. Andersen, BRIC Center, Copenhagen) was cultured in DMEM (Sigma–Aldrich) plus 10% fetal bovine serum (FBS).

For gene expression and microRNA analysis, MT-CHC01 cells (600,000/well) were plated onto 6-well tissue culture plates in complete Knockout/DMEM/F-12 medium plus 10% FBS; after 24 hours, they were treated with a dose of 5 nM of trabectedin, then, after further 24 hours, cells were detached and lysed in TRIZOL reagent for RNA extraction.

For cell viability assay, MT-CHC01 cells (3,000/well) were seeded onto 96-well tissue culture plates; after 24 hours they were treated with escalating doses of trabectedin (0.019-10 nM) in appropriate culture medium added of 10% FBS for another 72 hours. Cell growth was evaluated with the Cell Titer-Glo® cell viability assay (Promega). All tests were performed in quadruplicate and repeated in three independent experiments. IC50 values, dose of drug that inhibits 50% of the cell growth compared with control calculated for each cell line after 72 hours of drug treatment, were calculated using the CalcuSyn software, based on the Chou-Talalay method. To test the effect of the drug on cell cycle and apoptosis, MT-CHC01 cells were treated with 5 nM of trabectedin for 24-48 hours and then processed as described in our previous work [19].

### Gene and microRNA expression analysis

For gene expression analysis (GEP), total RNA of MT-CHC01s cells and of PDX, both untreated or treated with trabectedin, was extracted by using the Absolutely RNA miRNA kit (Agilent Technologies), following manufacturers’ protocols. Quantitative and qualitative evaluation of total RNA was performed by Nanodrop and BioAnalyzer respectively. For GEP analysis, 100 ng of total RNA were amplified and labeled using Low Input Quick Amp Labeling Kit, one-color kit (Agilent Technologies). Six hundred ng of labeled RNA were hybridized on SurePrint G3 Human Gene Expression 8x60K v2 glass arrays. Arrays were scanned and images analyzed by the Feature Extraction Software from Agilent Technologies (version 10.7), and raw data were then processed using the Bioconductor package Limma (Linear models for microarray analysis). Background correction was performed with the *normexp* method with an offset of 50, and *quantile* was used for the between-array normalization. The empirical Bayes method was used to compute a moderated t-statistics. For microRNA analysis, 100 ng of total RNA were labeled using the miRNA Complete Labeling and Hyb Kit and hybridized on Human miRNA Microarray Kit Release 16.0, 8x60K. Arrays were scanned and images analyzed by the Feature Extraction Software from Agilent Technologies (version 10.7). Raw data elaboration was carried out with Bioconductor, using R statistical language. *Quantile* was used for between-array normalization. The Limma (LInear Models for Microarray Analysis) package was then used to identify differentially expressed micro RNAs in trabectedin-treated versus not treated mice or cell line. The empirical Bayes method was used to compute a moderated t-statistics. Gene expression profiling and microRNA data are available on GEO (GSE84939).

### qRT-PCR validation

RNA was reverse-transcribed to cDNA with the High capacity cDNA reverse transcription kit (Applied Biosystem). The cDNA was used for amplification of CDH2, WNT7B, PMEPA1, NAV2, ATF3, NOV, CD68, RASD, SYK, LGALS1 deregulated genes and PGK housekeeping gene with specific primers (Supplementary Table S7). Quantitative real-time PCR was carried out in triplicate. Further, a validation of the expression of miR-4284, miR 21-5p, and miR-494-3p was performed using TaqMan Assay. MicroRNAs expression was normalized on the expression of RNU48 as endogenous control. Quantitative analysis was performed by the measurement of Ct values [57].

### Gene silencing

About 600,000 MT-CHC01 and WITT cells were plated in a 6 multiwell plate; at about 50% of confluence, cells were transiently transfected using oligofectamine (Life Technologies) and specific siRNAs for LGALS1, SYK, their combination, and negative control siRNA (all from Sigma) at the concentration of 20 μM in Optimum medium without serum. After 4 hours, cells were washed and complete and appropriate medium for each cell line was replaced. After 24 hours, the SYK and LGALS1 silencing efficiency was tested by qRT-PCR and Western blot analysis. Briefly, cells were detached and lysed in TRIZOL for RNA extraction and qRT-PCR analysis as previously described, and in Cell lysis buffer (Cell Signaling Technology, Beverly, USA) for Western blot analysis; homogenized cells were then centrifuged at 20,000x*g* for 30 minutes; 20 μg of protein were separated with Mini-Protean TGX Precast Gels, 4-20%, then transferred using Trans-Blot Turbo on nitrocellulose Midi membranes (Biorad). Blots were stained using standard procedures and signals were revealed by a chemiluminescence reagent (Euroclone, Milan, Italy). Primary antibody against LGALS1, SYK, Vinculin, and secondary anti-rabbit and anti-mouse IgG hrp-linked antibodies are from Cell Signaling.

### Wound healing assay

After 24 hours from silencing, the confluent MT-CHC01 and WITT cells were gently wounded with a 200 μl tip; wound closure was monitored in each plate for 24 hours. The gap distance was analyzed using ImageJ software; 0% represents the time of wound (T0). The percentage of closure was calculated as 100-(T/T0) x 100, where T represents the average wound closures at the different time points. Statistical analysis was performed using one-way ANOVA and multiple comparison test (GraphPad software). The assay was performed in three different experiments.

## SUPPLEMENTARY FIGURES AND TABLES








