# Supplementary material for: Gene and microRNA modulation upon trabectedin treatment in a human intrahepatic cholangiocarcinoma paired patient derived xenograft and cell line
Source: Oncotarget. 2016 Nov 24;7(52):86766–80. doi: 10.18632/oncotarget.13575 (PMC5349952; doi:10.18632/oncotarget.13575)
Supplement: Supplementary file 1 [file oncotarget-07-86766-s001.pdf]

Gene and microRNA modulation upon trabectedin treatment in a human intrahepatic cholangiocarcinoma paired patient derived xenograft and cell line

SUPPLEMENTARY FIGURES AND TABLES

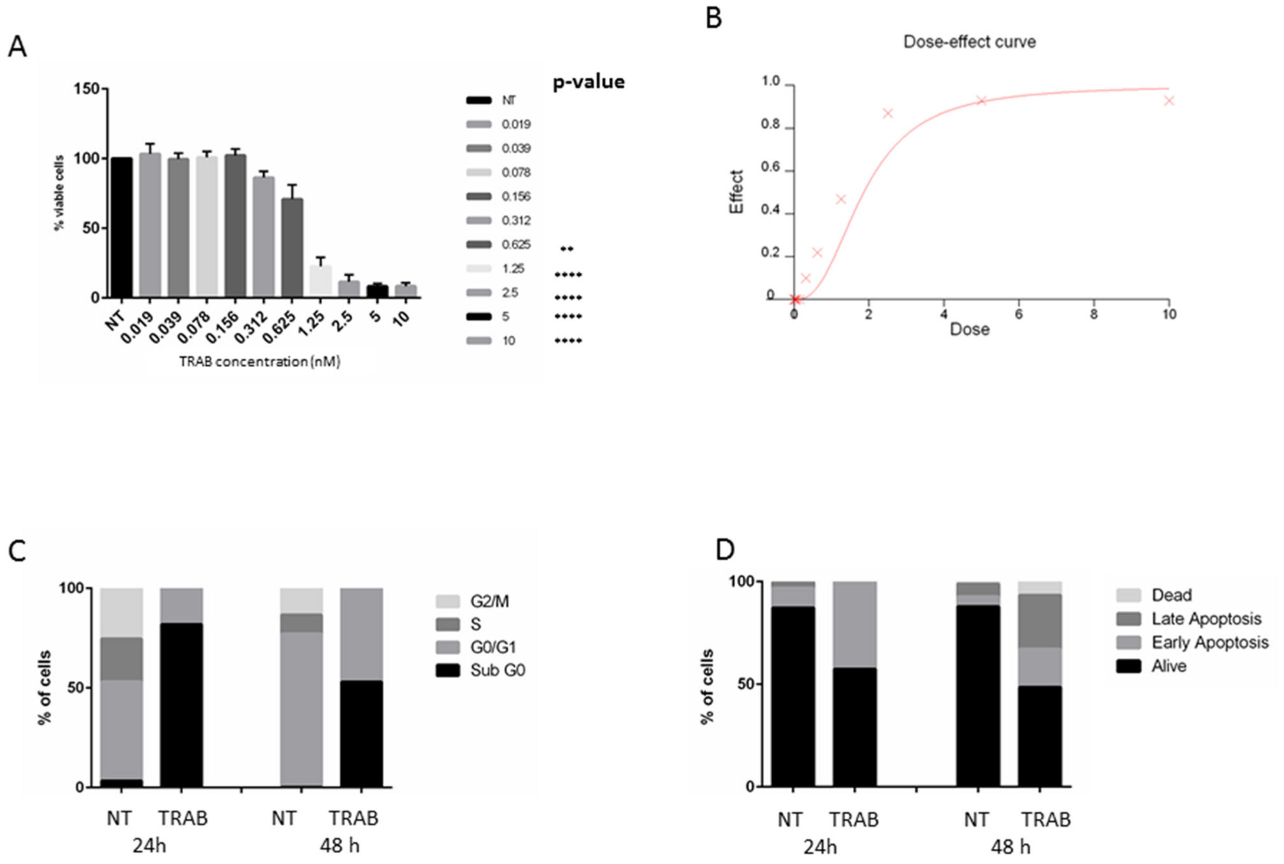

Supplementary Figure S1: Effect of trabectedin on proliferation, cell cycle and apoptosis in MT-CHC01 cells. Panel A: Statistical analysis of cell viability after 72 hours of treatment with escalating doses of trabectedin. A statistical significant reduction of proliferation was revealed until 0.625 nM. **Panel B**: Dose effect curve of trabectedin; the IC50 value is 1.84 nM. **Panels C-D**: Effect of trabectedin on cell cycle (C) and on apoptosis (D) *in vitro*. MT-CHC01 cells were treated with 5 nM of trabectedin for 24 and 48 h and subjected to cell cycle analysis and apoptosis detection by AnnexinV/PI staining by flow cytometry as described in methods. The bars represent the average with SEM of percentage of cells in each phase (C) and apoptotic cells (D) of three independent experiments. NT: not treated cells

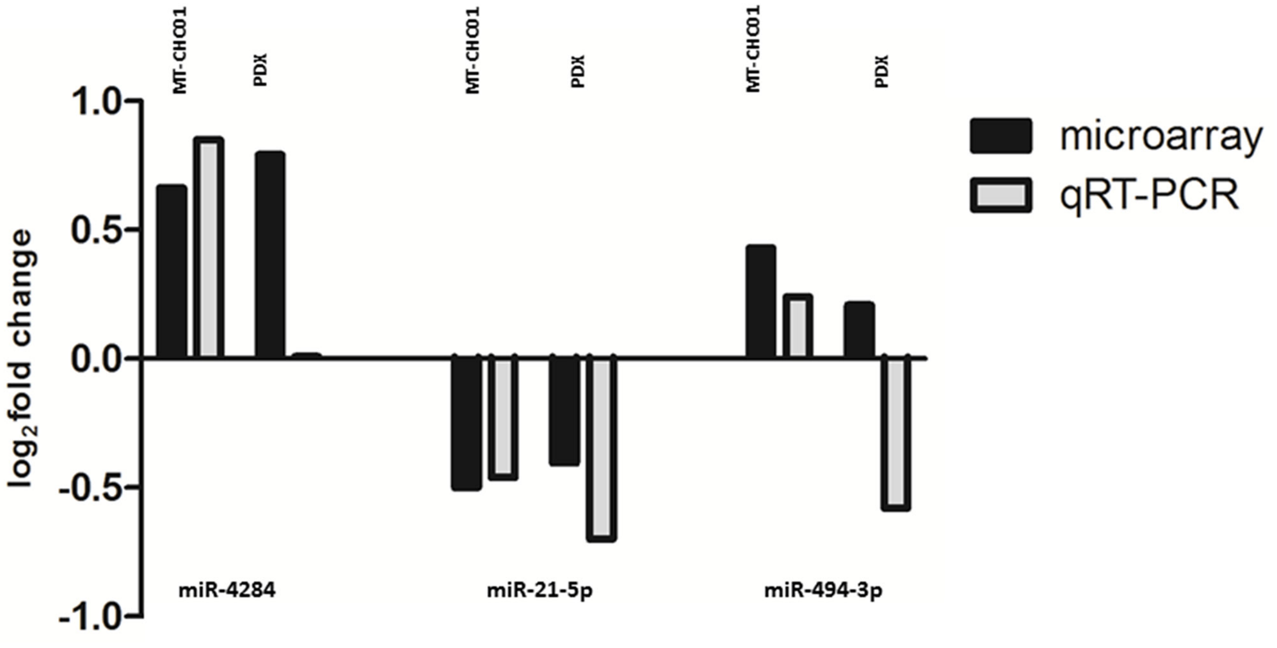

Supplementary Figure S2: Comparison between the expression values of selected microRNAs obtained by array and qRT-PCR after trabectedin treatment. X axis: gene expression of MT-CHC01 and PDX respectively. Y axis: $\log_2$ fold change expression obtained by microarray and $2^{-\Delta\Delta}$Ct for qRT-PCR.

Supplementary Table S1: Biological processes significantly enriched within down- and up- regulated probes after trabectedin treatment of MT-CHC01 cells.

See Supplementary File 1

Supplementary Table S2: Pathways enriched within down- and up-regulated genes (Kegg database) after trabectedin treatment of MT-CHC01 cells

Name	p-value	Expression upon trabectedin
**Focal adhesion**	0.000001	down
**Fc gamma R-mediated phagocytosis**	0.001	down
**Endocytosis**	0.001	down
**Regulation of actin cytoskeleton**	0.003	down
**Tight junction**	0.009	down
**Glioma**	0.01	down
**O-Glycan biosynthesis**	0.01	down
**Axon guidance**	0.01	down
**ErbB signaling pathway**	0.03	down
**Fc epsilon RI signaling pathway**	0.04	down
**Adherens junction**	0.04	down
**Insulin signaling pathway**	0.04	down
**Cytokine-cytokine receptor interaction**	0.01	up
**Pathways in cancer**	0.02	up
**p53 signaling pathway**	0.03	up

Supplementary Table S3: Biological processes significantly enriched within down and up regulated probes after trabectedin treatment of PDX

See Supplementary File 2

Supplementary Table S4: Pathways enriched within down- and up-regulated genes (Kegg database) after trabectedin treatment of PDX

Name	p-value	Expression upon trabectedin
Focal adhesion	0.001	down
ECM-receptor interaction	0.002	down
Complement and coagulation cascades	0.001	down
Systemic lupus erythematosus	0.004	down
Basal cell carcinoma	0.008	down
Arrhythmogenic right ventricular cardiomyopathy (ARVC)	0.03	down
Hedgehog signaling pathway	0.04	down
MAPK signaling pathway	0.02	up
Inositol phosphate metabolism	0.02	up
Complement and coagulation cascades	0.02	up

Supplementary Table S5: Common deregulated genes between *in vitro* and *in vivo* models upon trabectedin treatment

See Supplementary File 3

Supplementary Table S6: Differentially expressed microRNA upon trabectedin of MT-CHC01 cells

miRNA	logFC	Function	PMID
**hsa-miR-27b-3p**	-0.56	5-FU resistance	25356050
**hsa-miR-28-3p**	-0.56	Migration/invasion	22240480
**hsa-miR-628-5p**	-0.55	Sunitinib resistance	24475095
**hsa-miR-21-3p**	-0.53	BTC progression	24699315/ 23417858/ 22213236
**hsa-miR-21-5p**	-0.50	BTC progression	22213236
**hsa-miR-210-3p**	-0-47	Hypoxia	24517586
**hsa-miR-331-3p**	-0.44	proliferation	24825302
**hsa-miR-31-3p**	-0.44	BTC progression	22213236
**hsa-miR-27a-3p**	-0.42	Hypoxia	24517586
**hsa-miR-200a-5p**	-0.40	Tumor progression	25275448
**hsa-miR-151a-3p**	-0.40	No cancer related	
**hsa-miR-29a-3p**	-0.39	Marker for AML NCS<br>Prognostic marker in NSCLC	21678057<br>24898736<br>24928469
**hsa-miR-574-5p**	0.39	Marker in ESCC<br>Tumor suppressor in CRC metastasis	23124769<br>24936152
**hsa-miR-3162-5p**	0.39	SCC progression marker	23799609
**hsa-miR-1915-3p**	0.40	Apoptosis	24814047/ 22121083
**hsa-miR-494-3p**	0.43	Proliferation/invasion	24644030
**hsa-miR-3656**	0.46		
**hsa-miR-3665**	0.46		
**hsa-miR-1268a**	0.57		
**hsa-miR-1207-5p**	0.61	Tumor progression	24481448
**hsa-miR-1225-5p**	0.66	No cancer related	
**hsa-miR-4284**	0.67	Apoptosis	24732116
**hsa-miR-1246**	0.71	Cervical cancer progression	24806621
**hsa-miR-4281**	0.79	Melanoma marker	23111773

Supplementary Table S7: Primers sequences for qRT-PCR validation

Gene Name	Forward	Reverse
CDH2	CCAGTATCCGGTCCGATCTG	ATAGTCCTGCTCACCACCAC
WNT7B	GAAGGAGAAGTACAACGCGG	ACACCAGGTCTGTCTCCATG
PMEPA1	ACCATCTTCGACAGTGACCT	GATGACCTCGCTGTAGGTGG
NAV2	AGTTACGGCCTGAGGATGTC	GTTGCTGTTGGAGTCGCTG
ATF3	TCGGAGAAGCTGGAAAGTGT	TTCTGAGCCCGGACAATACA
NOV	AGCTGTGGTATGGGGTTCTC	AGGTGGATGGCTTTGAGTGA
CD68	GCATCCCTTCGAGATCTCCA	GCAGGAGAAACTTTGCCCAA
RASD1	GCCAAGAAGAACAGCAGCC	AGTACTGCACCGAGACCTTG
SYK	AGCAAAAGCGATGTCTGGAG	ATCTCTCTTGGACACCCTGC
LGALS1	CTGTCTTTCCCTTCCAGCCT	GCGGTTGGGGAACTTGAATT
PGK	AGCTGCTGGGTCTGTCATCCT	TGGCTCGGCTTTAACCTTGT
