# Supplementary material for: Gene and microRNA modulation upon trabectedin treatment in a human intrahepatic cholangiocarcinoma paired patient derived xenograft and cell line
Source: Oncotarget. 2016 Nov 24;7(52):86766–80. doi: 10.18632/oncotarget.13575 (PMC5349952; doi:10.18632/oncotarget.13575)
Supplement: Supplementary file 2 [file oncotarget-07-86766-s002.docx]

**Supplementary Table 1. Biological processes significantly enriched within down and up regulated probes after trabectedin treatment of MT-CHC01 cells**.

| **Category**  **(BP-5)** | **Name** | **p-value** | **Expression upon**  **trabectedin** |
| --- | --- | --- | --- |
| **0051056** | regulation of small GTPase mediated signal transduction | 0.0000001 | down |
| **0006464** | protein modification process | 0.0000001 | down |
| **0043087** | regulation of GTPase activity | 0.0000001 | down |
| **0022008** | neurogenesis | 0.0000001 | down |
| **0048699** | generation of neurons | 0.000001 | down |
| **0048858** | cell projection morphogenesis | 0.000001 | down |
| **0048666** | neuron development | 0.000001 | down |
| **0016310** | phosphorylation | 0.000001 | down |
| **0000902** | cell morphogenesis | 0.000001 | down |
| **0032990** | cell part morphogenesis | 0.000001 | down |
| **0009966** | regulation of signal transduction | 0.000001 | down |
| **0044267** | cellular protein metabolic process | 0.000001 | down |
| **0051493** | regulation of cytoskeleton organization | 0.000001 | down |
| **0031175** | neuron projection development | 0.000001 | down |
| **0048812** | neuron projection morphogenesis | 0.000001 | down |
| **0016477** | cell migration | 0.000001 | down |
| **0010810** | regulation of cell-substrate adhesion | 0.001 | down |
| **0016197** | endosome transport | 0.001 | down |
| **0007507** | heart development | 0.001 | down |
| **0043242** | negative regulation of protein complex disassembly | 0.002 | down |
| **0060284** | regulation of cell development | 0.002 | down |
| **0031110** | regulation of microtubule polymerization or depolymerization | 0.002 | down |
| **0007409** | axonogenesis | 0.002 | down |
| **0000904** | cell morphogenesis involved in differentiation | 0.003 | down |
| **0007167** | enzyme linked receptor protein signaling pathway | 0.003 | down |
| **0007229** | integrin-mediated signaling pathway | 0.004 | down |
| **0031122** | cytoplasmic microtubule organization | 0.004 | down |
| **0009101** | glycoprotein biosynthetic process | 0.004 | down |
| **0035108** | limb morphogenesis | 0.004 | down |
| **0007417** | central nervous system development | 0.005 | down |
| **0007026** | negative regulation of microtubule depolymerization | 0.005 | down |
| **0031114** | regulation of microtubule depolymerization | 0.005 | down |
| **0001707** | mesoderm formation | 0.005 | down |
| **0007163** | establishment or maintenance of cell polarity | 0.005 | down |
| **0009142** | nucleoside triphosphate biosynthetic process | 0.006 | down |
| **0031111** | negative regulation of microtubule polymerization or depolymerization | 0.006 | down |
| **0048667** | cell morphogenesis involved in neuron differentiation | 0.006 | down |
| **0001952** | regulation of cell-matrix adhesion | 0.006 | down |
| **0048332** | mesoderm morphogenesis | 0.006 | down |
| **0051963** | regulation of synaptogenesis | 0.007 | down |
| **0050767** | regulation of neurogenesis | 0.008 | down |
| **0001704** | formation of primary germ layer | 0.009 | down |
| **0009144** | purine nucleoside triphosphate metabolic process | 0.009 | down |
| **0031424** | keratinization | 0.00001 | up |
| **0008544** | epidermis development | 0.003 | up |
| **0008637** | apoptotic mitochondrial changes | 0.004 | up |
| **0007398** | ectoderm development | 0.004 | up |
| **0009888** | tissue development | 0.004 | up |
| **0048754** | branching morphogenesis of a tube | 0.005 | up |
| **0009913** | epidermal cell differentiation | 0.005 | up |

BP-5: biological processes level 5
