# Supplementary material for: Gene and microRNA modulation upon trabectedin treatment in a human intrahepatic cholangiocarcinoma paired patient derived xenograft and cell line
Source: Oncotarget. 2016 Nov 24;7(52):86766–80. doi: 10.18632/oncotarget.13575 (PMC5349952; doi:10.18632/oncotarget.13575)
Supplement: Supplementary file 3 [file oncotarget-07-86766-s003.docx]

**Supplementary Table 3. Biological processes significantly enriched within down and up regulated probes after trabectedin treatment of PDX**.

| **Category**  **(BP-5)** | **Name** | **p-value** | **Expression upon trabectedin** |
| --- | --- | --- | --- |
| **0048593** | camera-type eye morphogenesis | 0.000001 | down |
| **0009888** | tissue development | 0.000001 | down |
| **0016525** | negative regulation of angiogenesis | 0.000001 | down |
| **0048592** | eye morphogenesis | 0.000003 | down |
| **0009887** | organ morphogenesis | 0.001 | down |
| **0002449** | lymphocyte mediated immunity | 0.001 | down |
| **0043589** | skin morphogenesis | 0.001 | down |
| **0006937** | regulation of muscle contraction | 0.002 | down |
| **0042476** | odontogenesis | 0.002 | down |
| **0051592** | response to calcium ion | 0.003 | down |
| **0010638** | positive regulation of organelle organization | 0.004 | down |
| **0007517** | muscle organ development | 0.006 | down |
| **0051271** | negative regulation of cell motion | 0.006 | down |
| **0045765** | regulation of angiogenesis | 0.006 | down |
| **0002253** | activation of immune response | 0.006 | down |
| **0007507** | heart development | 0.006 | down |
| **0014706** | striated muscle tissue development | 0.007 | down |
| **0007423** | sensory organ development | 0.009 | down |
| **0060537** | muscle tissue development | 0.009 | down |
| **0050801** | ion homeostasis | 0.009 | down |
| **0048730** | epidermis morphogenesis | 0.009 | down |
| **0001654** | eye development | 0.009 | down |
| **0002526** | acute inflammatory response | 0.001 | up |
| **0060537** | muscle tissue development | 0.002 | up |
| **0007242** | intracellular signaling cascade | 0.002 | up |
| **0010558** | negative regulation of macromolecule biosynthetic process | 0.002 | up |
| **0009890** | negative regulation of biosynthetic process | 0.004 | up |
| **0014706** | striated muscle tissue development | 0.005 | up |
| **0009966** | regulation of signal transduction | 0.006 | up |
| **0016481** | negative regulation of transcription | 0.006 | up |
| **0031327** | negative regulation of cellular biosynthetic process | 0.007 | up |
| **0010556** | regulation of macromolecule biosynthetic process | 0.007 | up |
| **0008285** | negative regulation of cell proliferation | 0.007 | up |
| **0007517** | muscle organ development | 0.008 | up |
| **0030336** | negative regulation of cell migration | 0.009 | up |
| **0007507** | heart development | 0.009 | up |

BP5: Biological process level 5
